# MicroRNA-135b Regulates Leucine Zipper Tumor Suppressor 1 in Cutaneous Squamous Cell Carcinoma

**DOI:** 10.1371/journal.pone.0125412

**Published:** 2015-05-04

**Authors:** Edit B. Olasz, Lauren N. Seline, Ashley M. Schock, Nathan E. Duncan, Argelia Lopez, Jozef Lazar, Michael J. Flister, Yan Lu, Pengyuan Liu, Olayemi Sokumbi, Catherine A. Harwood, Charlotte M. Proby, Marcy Neuburg, Zelmira Lazarova

**Affiliations:** 1 Department of Dermatology, Medical College of Wisconsin, Milwaukee, Wisconsin, United States of America; 2 Department of Physiology, Medical College of Wisconsin, Milwaukee, Wisconsin, United States of America; 3 Centre for Cutaneous Research, Blizard Institute, Barts and the London School of Medicine and Dentistry, Queen Mary University of London, London, United Kingdom; 4 Division of Cancer Research, Medical Research Institute, Ninewells Hospital & Medical School, University of Dundee, Dundee, United Kingdom; University of Alabama at Birmingham, UNITED STATES

## Abstract

Cutaneous squamous cell carcinoma (cSCC) is the second most common skin malignancy and it presents a therapeutic challenge in organ transplant recipient patients. Despite the need, there are only a few targeted drug treatment options. Recent studies have revealed a pivotal role played by microRNAs (miRNAs) in multiple cancers, but only a few studies tested their function in cSCC. Here, we analyzed differential expression of 88 cancer related miRNAs in 43 study participants with cSCC; 32 immunocompetent, 11 OTR patients, and 15 non-lesional skin samples by microarray analysis. Of the examined miRNAs, miR-135b was the most upregulated (13.3-fold, 21.5-fold; *p*=0.0001) in both patient groups. Similarly, the miR-135b expression was also upregulated in three cSCC cell lines when evaluated by quantitative real-time PCR. In functional studies, inhibition of miR-135b by specific anti-miR oligonucleotides resulted in upregulation of its target gene LZTS1 mRNA and protein levels and led to decreased cell motility and invasion of both primary and metastatic cSCC cell lines. In contrast, miR-135b overexpression by synthetic miR-135b mimic induced further down-regulation of LZTS1 mRNA *in vitro* and increased cancer cell motility and invasiveness. Immunohistochemical evaluation of 67 cSCC tumor tissues demonstrated that miR-135b expression inversely correlated with LZTS1 staining intensity and the tumor grade. These results indicate that miR-135b functions as an oncogene in cSCC and provide new understanding into its pathological role in cSCC progression and invasiveness.

## Introduction

Cutaneous squamous cell carcinoma (cSCC) is the second most common human skin cancer, and it is reported to be within the top five most costly cancers in the US Medicare population [1]. Uncomplicated sporadic cSCCs are typically curable by surgery or a combination of surgery, topical chemotherapy, and radiotherapy. The clinical problem of cSCC is especially severe in immunocompromised individuals such as solid-organ transplant recipients (OTR). These patients have 65 to 250 times the normal incidence of cSCC and more importantly a significantly higher risk of metastasis [2]. Metastatic cSCC present a significant therapeutic problems since chemotherapy is not consistently efficient [3]. In addition, prognosis for OTR patients with cSCC is extremely poor, with an overall survival rate at 1 year of 44–56% [3]. Thus, there is a great need to identify specific molecules involved in cSCC invasion and metastasis which can potentially serve as target for new treatment strategies.

MicroRNAs (miRNAs) are small non-coding RNA molecules capable of regulating gene expression at the post-transcriptional level [4]. They can repress translation or induce mRNA cleavage by binding to the 3’ untranslated region of target mRNA [5, 6]. Alteration of miRNA expression has emerged as one of the key features in cancer-associated dysfunction of gene regulatory networks. Until recently only few studies have evaluated the miRNA expression in cSCC [7–11]. A previous microarray analysis of miRNA from immunocompetent (IC) cSCC patient-matched samples revealed changed expression of 9 miRNAs in cSCC tissues, of which miR-135b showed the largest change (upregulated 8.5-fold) between cancerous and paired normal tissues [8].

miR-135b has been implicated in cancer growth [12], survival [13], motility [14], and invasiveness [15] and has been shown to be upregulated in multiple other tumor types (i.e., breast, lung, colon, and prostate) [13, 15–17]. Interestingly, miR-135b function has been described as an oncogenic [14–16] or tumor-suppressive [18] in malignant tumors originated from different tissues. In head and neck SCC miR-135b was described as a tumor promoter by stimulating cancer cell proliferation, colony formation, and angiogenesis through activation of HIF-1α [19]. However, despite miR-135b being upregulated in cSCC, the function of this miRNA in the cSCC progression has not been investigated.

The leucine zipper tumor suppressor 1 (LZTS1) gene was described as the tumor suppressor gene by Ishii at all in 1999 [20]. Functional studies demonstrated that deletion of one or both alleles of LZTS1 gene in mouse results in onset of spontaneous tumors in several organs including skin [21] and reintroduction of LZTS1 gene suppresses tumorigenicity in nude mice *in vivo* [22]. Recently LZTS1 has been identified as a target-gene of miR-135b [15] and has been implicated in tumor growth [23], motility [24], and invasiveness [22] in multiple tumors. However, the role of LZTS1 in cSCC progression and the clinical implication between miR-135b expression, LZTS1 expression and histological tumor grade has not yet been tested.

In this study we tested the expression of miR-135b and LZTS1 in cSCC tumor tissues obtained from IC and OTR patients and cSCC cell lines. Keratinocyte cell lines spontaneously derived from human skin are rare and published cSCC lines usually represent a late stage of malignant transformation [25]. To evaluate the miR-135b regulation of LZTS1 expression *in vitro* we have used three cSCC cell lines derived from an immunosuppressed renal transplant (OTR) patient described and characterized previously [26, 27]. These unique cell lines represent early and advanced stages of malignant transformation of the epidermis starting from dysplastic (PM1), to primary tumor (MET1), and metastatic tumor (MET4) and provide a tool for evaluation of microRNA expression at the different stage of disease progression *in vitro*. Using multiple gain-of-function and loss-of-function assays, we describe for the first time that LZTS1 is indeed a target gene of miR-135b in cSCC and mirR-135b expression inversely correlates with staining intensity of LZTS1and tumor grade in cSCC tumor samples. In addition, inhibition of miR-135b blocks cSCC cell motility and invasiveness without inhibition of cell proliferation. Collectively, our data demonstrate that miR-135b plays an oncogenic role in cSCC through downregulation of tumor suppressor gene LZTS1and may potentially serve as a molecular target for therapeutic interventions in OTR patients with multiple or difficult to manage tumors.

## Materials and Methods

### Clinical samples

Tissue samples were taken after obtaining written informed consent from cSCC patients undergoing Mohs-micrographic surgery at the Skin Cancer Center, Froedtert Hospital and Medical College of Wisconsin, Milwaukee. This study was approved by the Medical College of Wisconsin Institutional Review Board and in accordance with the Declaration of Helsinki Principles (Institutional Review Board Assurance of Compliance Number for this study: FWA00000820). The clinical diagnosis of cSCC was made by the Mohs-surgeon and tumor grade and LZTS1 staining intensity was evaluated and confirmed by dermatopathologists. Tumor tissue was obtained from 134 patients as follows: A) 32 IC patients, 11 OTRs patients (total n = 43), 15 non-lesional skin samples for miRNA microarray studies. B) 42 paraffin-embedded and 25 OCT-embedded tumor samples for immunohistochemistry studies.

### Cancer cell lines

The human cSCC cell lines MET1 and MET4 were derived from a single renal transplant recipient (OTR) patient who suffered from multiple cSCCs. MET1 was derived from a primary invasive cSCC tumor and MET4 from an associated lymph node metastasis [26]. The PM1 keratinocyte cell line was cloned from cultures of dysplastic forehead skin of the same patient that showed an extended life span *in vitro*. These cell lines represent the first sequential series of cSCC from the same patient that can be used as a model to study the course of epigenetic regulations *in vitro* [26]. All cell lines were thawed from frozen stocks, cultured in a nutrient mix of Dulbecco’s minimal essential medium with Ham’s F12 medium (3:1) supplemented with hydrocortisone, human insulin, cholera toxin, Apo-transferrin, lyothyronine (L4), and antibiotics/antimycotics in a 37°C incubator at 5% CO_2_.

### Real-time PCR array analysis of miRNA expression

Total RNA, including miRNA, was isolated with the *mir*Vana miRNA Isolation Kit (Life Technologies, Grand Island, NY) from all tumor and control non-lesional skin samples. The RNA concentration and purity were determined using the NanoDrop ND-2000 spectrophotometer (Thermo Fisher Scientific, Waltman, USA). Tissue samples were analyzed for the presence and differential expression of a panel of 88 cancer-related miRNAs using a human cancer specific RT^2^ miRNA PCR array (SABiosciences) according to the manufacturer's instructions. The miRNA expression was normalized to four housekeeping genes (SNORD48, SNORD47, SNORD44, RNU6-2) and then tumor miRNA levels were compared with those in non-lesional skin samples using manufacturer's software. Resulting miRNA expression data was submitted to the ArrayExpress database (http://www.ebi.ac.uk/arrayexpress/) with the accession number of E-MTAB-3409.

### Transfections assay

Cells were transfected with 10nM of *mir*Vana inhibitor or 15nM of *mir*Vana mimic to miR-135b (Life Technologies) using Lipofectamine RNAiMAX (Life Technologies) according to the manufacturer’s protocol. For each set of cells a corresponding *mir*Vana negative control for the inhibitor or mimic was used. qRT-PCR analysis of LZTS1 mRNA expression in the transfected cells was performed with High Capacity RNA-to-cDNA Kit (Life Technologies) and Power SYBR Green Master Mix (Life Technologies). Primers used for qRT-PCR amplification of human LZTS1 gene were as follows; Fwd 5’-ACCTCTAGAAACCCAGAACTCA-3’, Rev 5’-TCCAGAAGAGCCCATATCACTA-3’. The results were normalized to the levels of the housekeeping gene GAPDH; Fwd 5’-GCGCCCAATACGACCAA-3’, Rev 5’-CTCTCTGCTCCTCCTGTTC-3’. Gene transcripts were amplified by qRT-PCR using the Applied Biosystems StepOnePlus cycler. Thermal cycler conditions were as follows: 2 minute UNG incubation at 50°C and 10 minute polymerase activation at 95°C followed by forty cycles of 95°C for 15 sec and 60°C for 1 minute.

### Migration assay

To evaluate cSCC cell line motility, transfected cells were first grown to confluency. The monolayer was scratched in a standard manner using a 200 μl sterile pipette tip to create a cell-free zone. The medium was aspirated and replaced with fresh complete medium then the cells were incubated for 24 hours at 37°C. *In vitro* migration was documented at 24 hours by photography. The assay was performed in triplicate for each experimental condition. The residual gaps between the migrating keratinocytes were measured at five random intervals for each experimental condition and then expressed as a percentage of the original scratch width using NIH Image J program 1.48b.

### Transwell invasion assay

The invasion assay was performed using a 24-well BD BioCoat Invasion Chamber (BD Biosciences, USA). Cells transfected with 10nM of *mir*Vana inhibitor or 15nM of *mir*Vana mimic to miR-135b and appropriate controls were harvested 24 hours after transfection. The cells were re-suspended in a serum free media with reduced growth factors (as previously stated) and added to the upper chamber at a density of 1x10^4^ cells per well in triplicate; the bottom chamber contained growth media supplemented with 10% fetal bovine serum (FBS) and normal concentration of growth factors. After incubating for 24 hours, cells in the upper well were removed by aspiration of the Matrigel and wiping the top of the membrane with a cotton swab. Cells on the underside of the membrane were fixed using the Differential Quik Stain Kit (Polysciences, Inc., USA). Briefly, the membranes were submerged sequentially in each solution (A, B, and C) for 2 minutes, then rinsed twice in deionized water and allowed to air dry. Invading cells were evaluated using images of five microscopic fields per membrane under light microscopy at a magnification of 40x. The number of invading cells in each photograph were then counted using NIH Image J program 1.48b.

### Proliferation assay

To monitor cell proliferation of PM1, MET1, MET4 cell lines, 5000 cells/well were seeded in 96-well microplate in a final volume of 100ul. For proliferation assays we used the CyQUANT NF Cell proliferation Assay Kit (Invitrogen, USA) constructed on measurement of cellular DNA content via fluorescent dye binding. Briefly, the cells transfected with 10nM of *mir*Vana inhibitor or 15nM of *mir*Vana mimic to miR-135b and appropriate controls were incubated with CyQUANT NF reagent for 60 minutes at 37°. Fluorescence intensities were measured with a microplate reader using excitation at 485 and fluorescence detection at 530 nm at indicated time points according to manufacturer’s protocol. Each data point was obtained as an average of triplicate samples.

### Immunohistochemistry and immunocytochemistry

The LZTS1 protein expression was evaluated by immunohistochemistry on (n = 67) cSCC tumors. Twenty five tumor samples were embedded in OCT compound and processed by immunofluorescence studies, and forty two tumor samples were evaluated from paraffin embedded tissues. Frozen OCT-embedded 6-*μ*m-thick sections of cSCC were fixed for 10 minutes in 10% neutral buffered formalin then washed twice with phosphate buffered saline solution (PBS). Slides were blocked with 1% bovine serum albumin (BSA) in PBS for 1 hour at room temperature, then incubated with rabbit polyclonal antibody to LZTS1 (Novus Biologicals, USA) at a dilution of 1:200 for 1 hour at room temperature. Normal rabbit IgG at the same dilution served as a negative control. Following aspiration of the primary antibody and two washes in PBS, LZTS1 was detected by affinity-purified, Alexa Fluor 488 donkey anti-rabbit IgG (H+L) (Invitrogen, USA) at a dilution of 1:100 for 30 minutes. Slides were then washed and stained with DAPI (MP Biomedicals, USA) at a dilution of 1:1000 for one minute. At the end of the procedure, slides were washed, cover slipped, coded and evaluated under a fluorescent microscope. LZTS1 fluorescence staining intensity in the OCT-embedded tumor and non-lesional epidermis and in transfected cSCC cell lines was evaluated using images of five microscopic fields per slide at a magnification of 200x. Mean fluorescence intensity in five standardized areas of tumor and non-involved epidermis for each optical field were measured and the intensity of fluorescence staining was evaluated using the NIH Image J program 1.48b. The LZTS1 staining intensity in the cells transfected with miR-135b mimic or miR-135b inhibitor was evaluated after fixation of cells for 2 minutes with 95% ethanol in the same manner as the OCT-embedded tumor samples.

The paraffin-embedded tumor samples (n = 42) were deparaffinized and antigen retrieval was performed with Vector Antigen Unmasking Solution (Dako North America, Inc., USA). Slides were then blocked with 1% BSA in PBS plus 0.03% tween for 1 hour. Next, slides were stained using primary LZTS1 specific rabbit polyclonal antibody at 1:200 dilution overnight at 4°C. The Dako Envision system-HRP Kit was then used for the secondary HRP-labeled anti-rabbit IgG for 30 minutes at room temperature and developed with Chromogen Substrate (Dako Envision System-HRP kit). The slides were counterstained with hematoxylin (Vector Laboratories Inc., USA). The histological tumor grade was established by dermatopathologist using hematoxylin and eosin stained tumor sections as described previously [28]. The LZTS1 staining intensity was quantified in the paraffin-embedded tumor and non-lesional epidermis by dermatopathologist using semiquantitative grading scale where 0 represented absence of staining, 1 = light staining, 2 = moderate staining, and 3 = dark staining typically seen in the uninvolved normal epidermis which served as an internal control for each slide.

### Statistical analysis

For the microarray evaluation, the expression of miRNAs for all tumor samples was first normalized to four housekeeping genes and then compared with those in non-lesional skin samples. To assess the differences between the groups, nonparametric permutation tests were performed to calculate the *p*-value for each miRNA. False discovery rate was controlled for multiple testing [29]. The *p*-value of permutation less than 0.003 was considered statistically significant. To test the strength of the relationship between the tumor grade and LZTS1 staining intensity we used Spearman’s Rank correlation. For the remaining experiments the statistical analysis was performed using the Student *t* test. The data were expressed as mean ± standard deviation, the significance level was *p<0*.*05*.

## Results

### MicroRNA expression signatures in cSCC tissues

**Fig 1:**
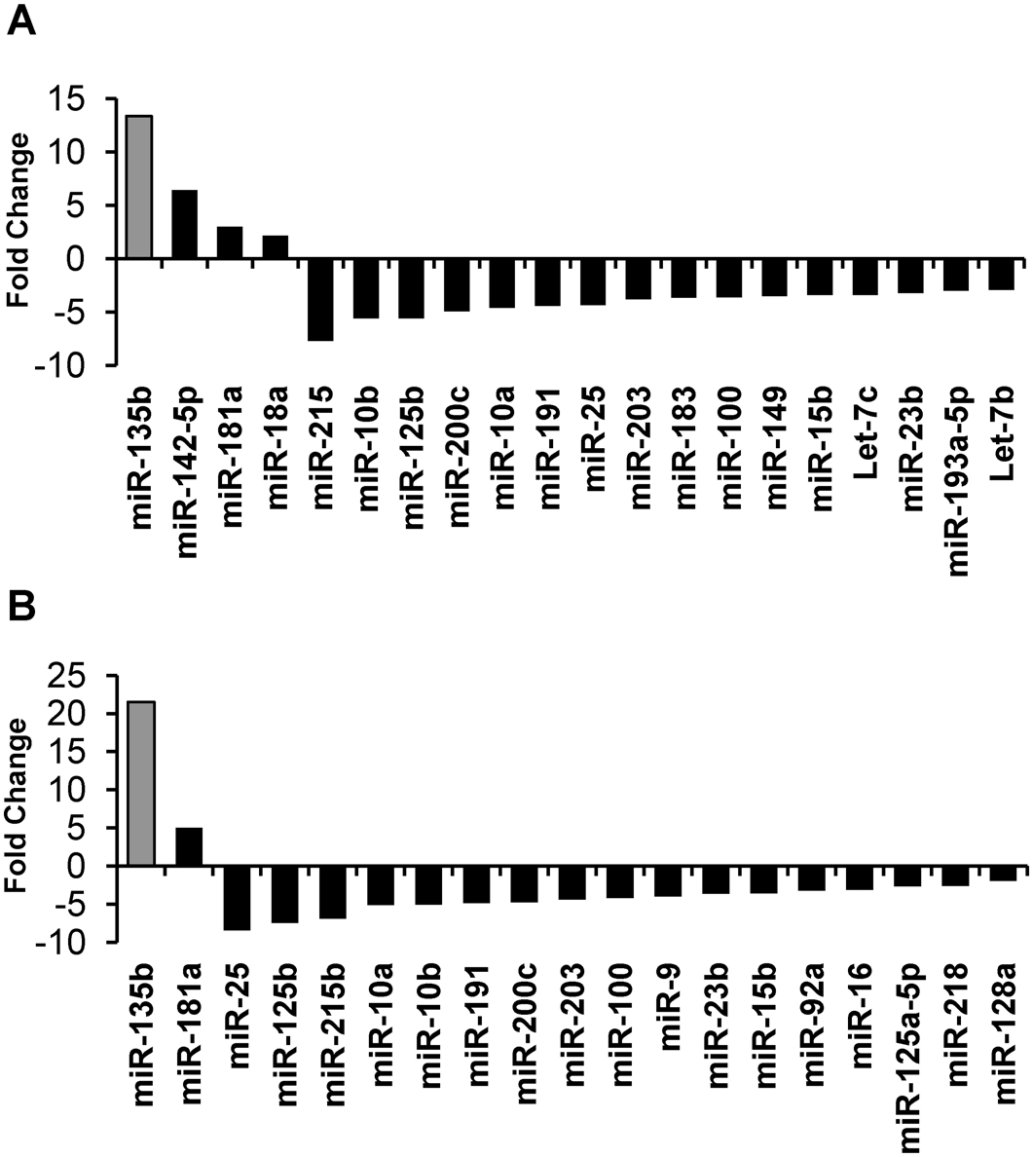
Differentially expressed miRNAs in human cSCC tissue. A) PCR array analysis of miRNA expression in cSCC tumors from immunocompetent patients (n = 32). B) PCR array analysis miRNA expression in cSCC tumors from organ transplant recipients (n = 11).

To test miRNA expression in cSCC, miRNA transcripts were isolated from cSCC samples of 32 IC patients, 11 OTRs patients (total n = 43), and control non-lesional skin samples (n = 15) and analyzed by qRT-PCR miRNA array. Compared with control samples, 4 miRNAs (miR-135b, miR-142-5p, miR-181a, and miR-18a) were significantly upregulated in cSCC from IC patients, whereas 16 were significantly downregulated in IC cSCC samples (Table 1). Two miRNA were significantly upregulated (miR-135b and miR-181a) and 17 were downregulated in cSCC samples from OTR patients (Table 2). Overall, we observed concordant changes in 11 of 26 differentially expressed genes between cSCC samples from IC and OTR patients. Of these genes, miR-135b had the largest difference in cSCC samples from OTR patients (21.5-fold; *p*<0.0001) and IC patients (13.3-fold; *p*<0.0001) compared with non-lesional controls (Fig 1A and 1B).

**Table 1:** Statistical analysis of differentially expressed miRNAs in cSCC tissues from immunocompetent patients.

miRNA ID	*p*-value of Permutation	Fold Change
hsa-miR-135b	*0*.*0001*	13.344
hsa-miR-142-5p	*0*.*0001*	6.41
hsa-miR-181a	*0*.*0003*	2.98
hsa-miR-18a	*0*.*0009*	2.162
hsa-miR-215	*0*.*0024*	-7.723
hsa-miR-10b	*0*.*0028*	-5.6
hsa-miR-125b	*0*.*0001*	-5.596
hsa-miR-200c	*0*.*0001*	-4.934
hsa-miR-10a	*0*.*001*	-4.605
hsa-miR-191	*0*.*0001*	-4.413
hsa-miR-25	*0*.*0017*	-4.368
hsa-miR-203	*0*.*0003*	-3.816
hsa-miR-183	*0*.*0035*	-3.659
hsa-miR-100	*0*.*0001*	-3.623
hsa-miR-149	*0*.*0013*	-3.51
hsa-miR-15b	*0*.*0001*	-3.408
hsa-let-7c	*0*.*0009*	-3.397
hsa-miR-23b	*0*.*0001*	-3.234
hsa-miR-193a-5p	*0*.*0012*	-2.988
hsa-let-7b	*0*.*002*	-2.925

**Table 2:** Statistical analysis of differentially expressed miRNAs in cSCC tissues from organ transplant recipients.

miRNA ID	*p*-value of Permutation	Fold Change
hsa-miR-135b	*0*.*0001*	21.496
hsa-miR-181a	*0*.*0005*	5.005
hsa-miR-25	*0*.*0001*	-8.447
hsa-miR-125b	*0*.*0001*	-7.445
hsa-miR-215	*0*.*0032*	-6.899
hsa-miR-10a	*0*.*0003*	-5.118
hsa-miR-10b	*0*.*0011*	-5.053
hsa-miR-191	*0*.*0001*	-4.849
hsa-miR-200c	*0*.*0001*	-4.757
hsa-miR-203	*0*.*0001*	-4.423
hsa-miR-100	*0*.*0001*	-4.21
hsa-miR-9	*0*.*0027*	-3.971
hsa-miR-23b	*0*.*0001*	-3.637
hsa-miR-15b	*0*.*0001*	-3.594
hsa-miR-92a	*0*.*0035*	-3.228
hsa-miR-16	*0*.*001*	-3.112
hsa-miR-125a-5p	*0*.*0017*	-2.657
hsa-miR-218	*0*.*0023*	-2.611
hsa-miR-128a	*0*.*0032*	-1.963

### LZTS1 expression is down-regulated in cSCC tissues and cell lines

**Fig 2:**
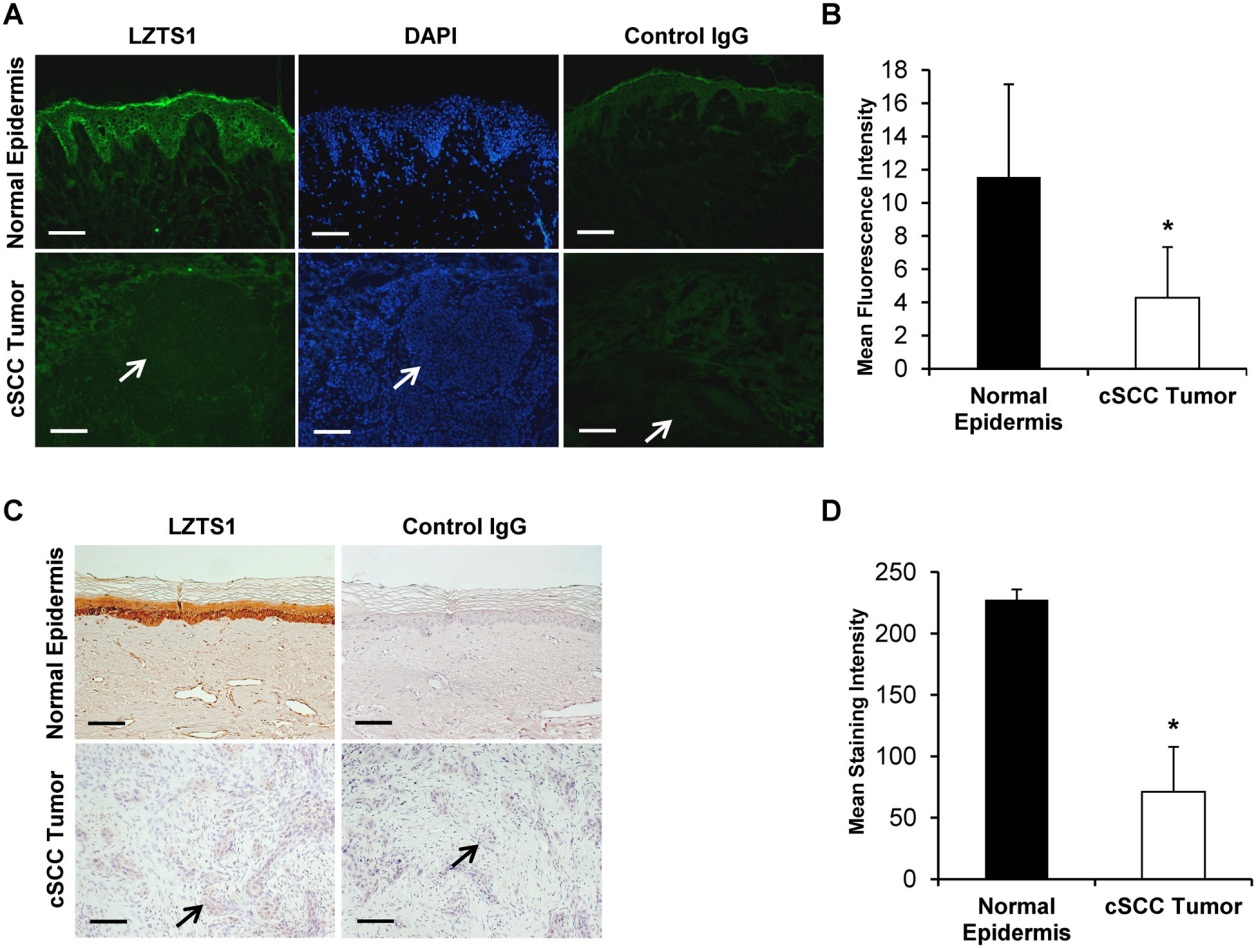
Immunohistochemical analysis of LZTS1 protein levels in normal human skin and cSCC tissues. A, C) LZTS1 staining is localized in the normal epidermis, but is largely absent in the tumor tissue. Arrows point towards the tumor nests. The absence of staining with control rabbit IgG antibody confirms the specificity of LZTS1 antibody. B, D) Evaluation of LZTS1 staining intensity shows significant reduction of LZTS1 staining in the tumor tissue when compared to normal non-lesional epidermis. * indicate p values less than 0.001. *Bar = 100um*.

**Fig 3:**
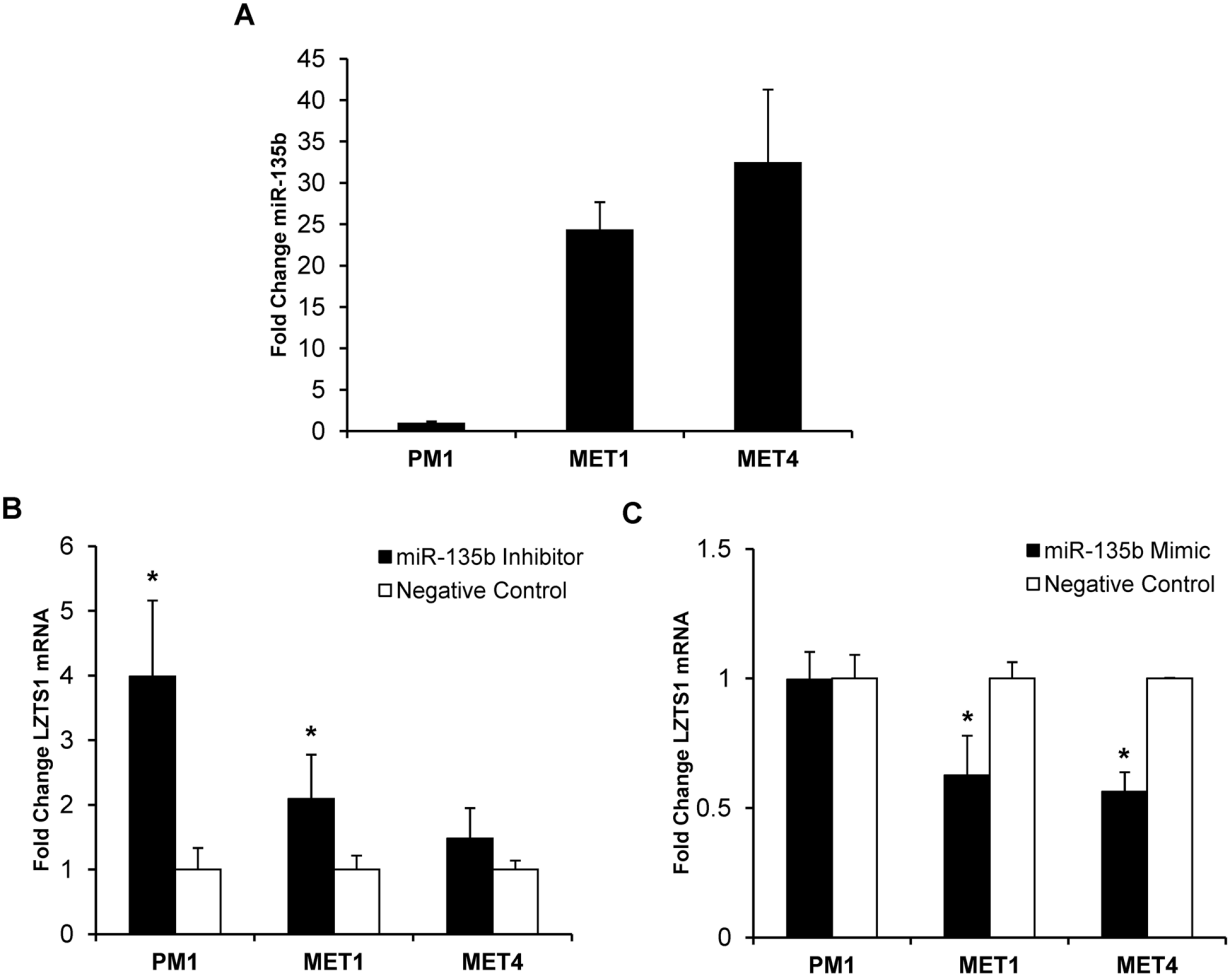
Functional assays of miR-135b inhibition and overexpression. A) Baseline mRNA miR-135b expression in 3 different OTR-derived cSCC cell lines was established by qRT-PCR. B) Effect of miR-135b inhibition on LZTS1 mRNA expression in cSCC lines by qRT-PCR within 48 hours post-transfection. C) Effect of miR-135b overexpression on LZTS1 mRNA expression in cSCC lines by qRT-PCR within 48 hours post-transfection. * indicate p values less than 0.005.

LZTS1 has recently been identified as a miR-135b target-gene in non-small-cell lung carcinoma that significantly correlates with patient survival [15]. In addition, down-regulation of LZTS1 is associated with poor prognosis in human breast carcinoma [24]. We hypothesized that miR-135b could also regulate LZTS1 expression in cSCC. To test this possibility, we first examined expression of LZTS1 protein expression by immunohistochemistry in cSCC (n = 25; 2 OTRs and 23 IC) and normal uninvolved skin matched samples. In normal skin, LZTS1 staining localized strongly to the basal and suprabasal layers with a mean fluorescent intensity (MFI) of 11.6± 5.6 (Fig 2A). In comparison, LZTS1 staining was markedly diminished in cSCC tumors (4.3 ± 3.1 MFI; *p*<0.001) (Fig 2A and 2B). LZTS1 staining intensity was also diminished in the 42 (8 OTRs and 34 IC) paraffin-embedded tumor samples (71.02 ± 36.5) when compared to non-lesional skin (227.5 ± 8.3; *p*<0.001) (Fig 2C and 2D). Importantly, the more invasive and less differentiated tumors (grades 3 and 4) had significantly lower LZTS1 staining intensity (r = -0.51, *p*<0.00058) then non-invasive or well differentiated tumors (grades 1 and 2); as estimated by Spearman’s rank correlation. No significant correlation was observed in LZST1 staining intensity and tumor grade between OTRs and IC samples. We detected reduced LZTS1 staining intensity in the tumor samples from OTRs. However, due to the low number of OTRs samples this trend did not reach statistical significance. Next we assessed miR-135b and LZTS1 expression in 3 human primary and metastatic cSCC cell lines by quantitative qRT-PCR. Expression of miR-135b was compared to the baseline expression in PM1 cells and RNU6B was used as a normalization control. Compared with PM1, miR-135b expression was significantly elevated in the primary invasive MET1 (24.4 ±2.3-fold; *p*<0.01) and metastatic MET4 (32.5 ± 8.9-fold; *p*<0.01) cell lines (Fig 3A). In line with our previous findings on the tumor tissues, the miR-135b expression was higher in the metastatic cell line (MET4) than in the primary invasive (MET1) cell line. Taken together, these data suggest an association between LZTS1 and miR-135b expression in cSCC.

### miR-135b regulates LZTS1 expression in cSCC cell lines

**Fig 4:**
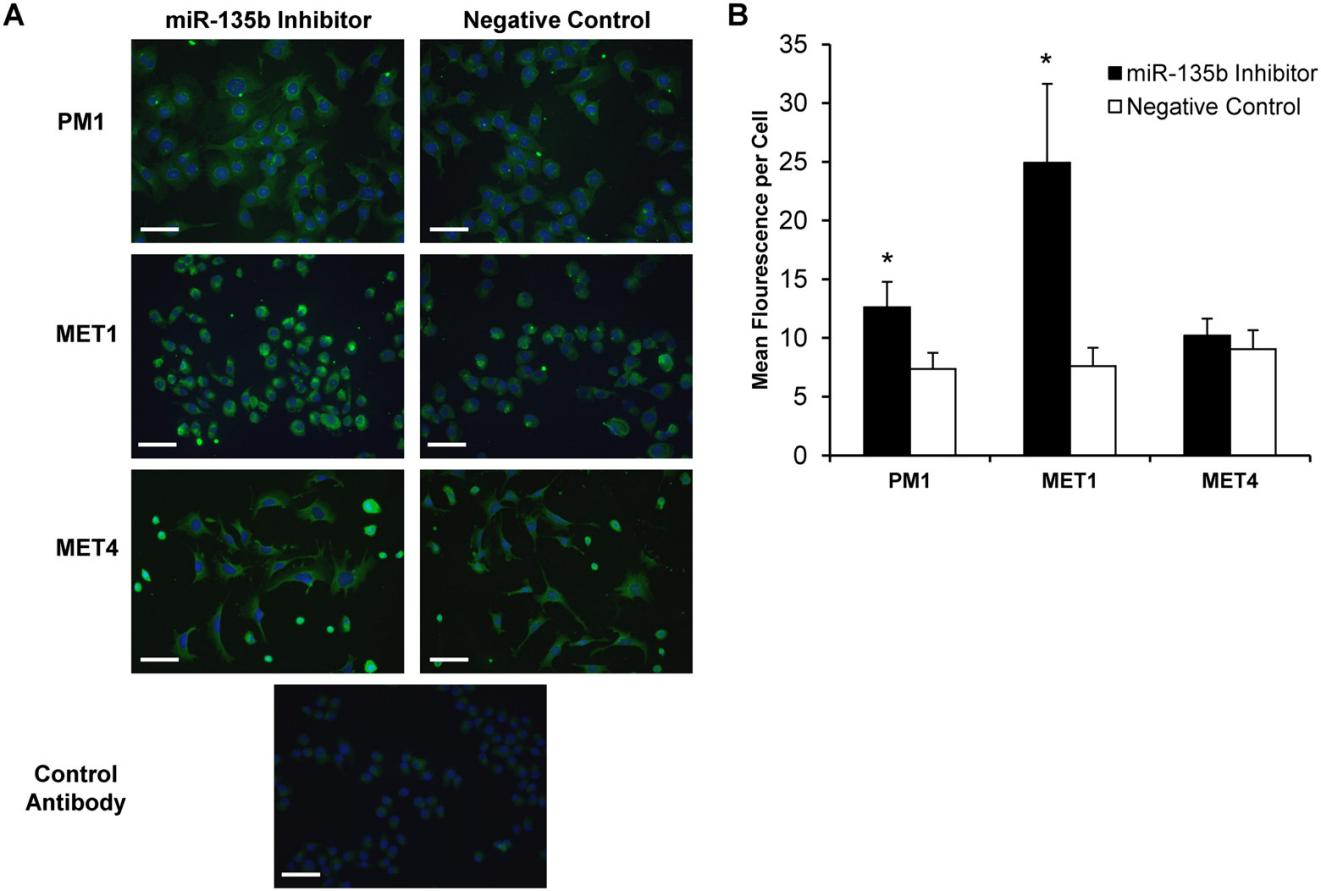
Immunohistochemical analysis of LZTS1 protein levels in cells transfected with miR-135b inhibitor. A) cSCC cell lines transfected with miR-135b inhibitor showed increased LZTS1staining intensity 48 hours post-transfection when compared to negative control. B) Mean fluorescent intensity per cell was quantified using NIH image J program in cell transfected with miR-135b inhibitor and negative control oligonucleotiides. * indicates p values less than 0.001. *Bar = 1000um*.

**Fig 5:**
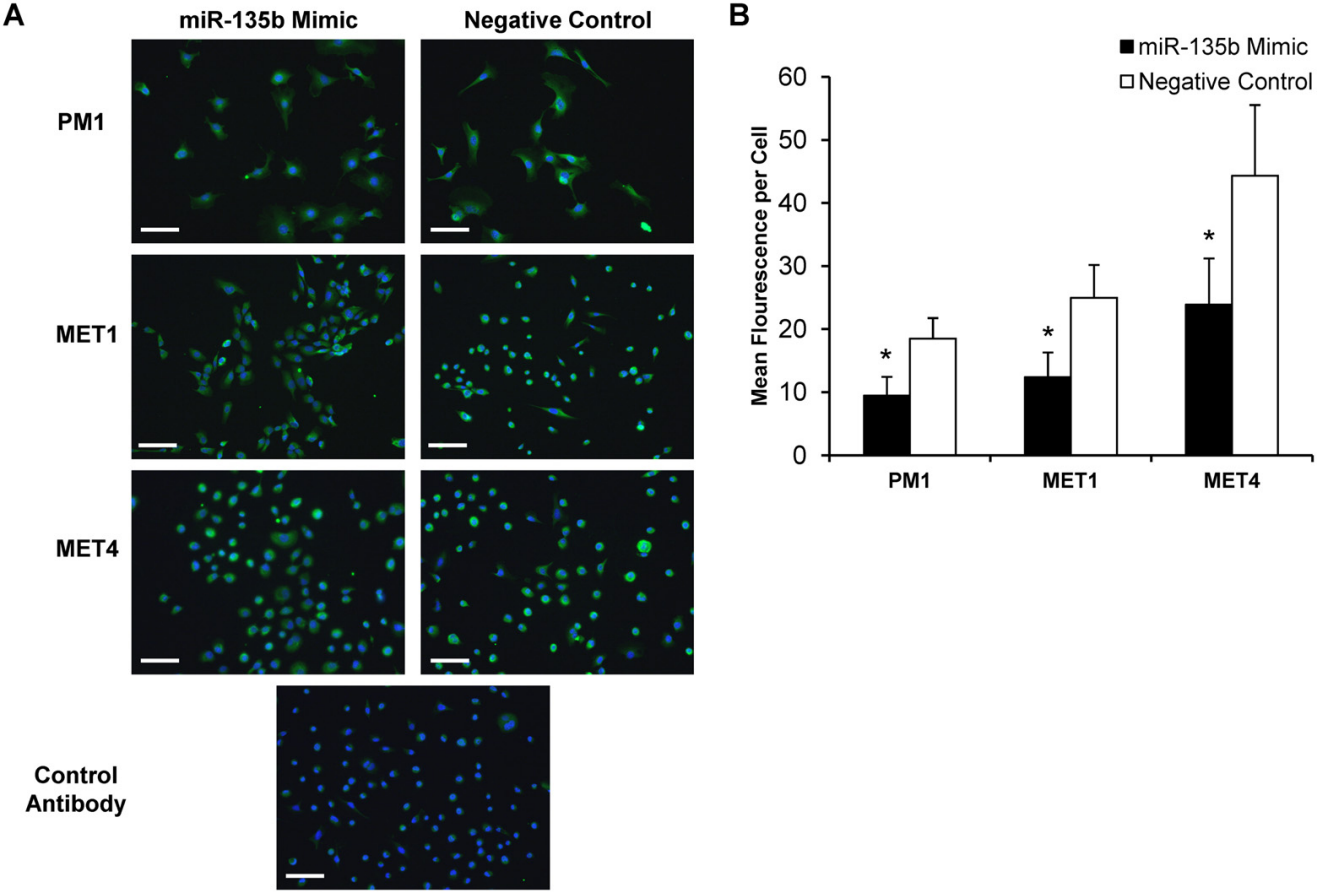
Immunohistochemical analysis of LZTS1 protein levels in cells transfected with miR-135b mimic. A) All cSCC cell lines transfected with miR-135b mimic showed decreased LZTS1staining intensity 48 hours post-transfection when compared to negative control. B) Mean fluorescent intensity per cell was quantified using NIH image J program in cell transfected with miR-135b mimic and negative control oligonucleotiides. * indicates p values less than 0.001. *Bar = 1000um*.

To directly test whether miR-135b regulates LZTS1 expression in cSCC *in vitro*, the PM1, MET1, and MET4 cell lines were transfected with synthetic *mir*Vana miR-135b inhibitor, *mir*Vana miR-135b mimic, or *mir*Vana negative control and tested in functional assays. At 48 hours post-transfection LZTS1 expression was assessed by qRT-PCR, and quantitative immunofluorescent staining of the cSCC cell lines. Compared with the negative control, the miR-135b inhibitor significantly increased LZTS1 mRNA expression in PM1 (2.1 ± 0.7-fold; *p* < 0.05), MET1 (4.0 ± 1.2-fold; *p*< 0.05), and to a lesser extent in MET4 (1.5 ± 0.5-fold) (Fig 3B). Similar to changes in LZTS1 mRNA transcript expression, LZTS1 staining intensity was significantly increased in PM1 (3.34 ± 2.0-fold; *p*<0.001) and MET1 (1.7 ± 0.7-fold; *p*<0.001) cells treated with the miR-135b inhibitor, whereas LZTS1 staining intensity in MET4 did not change (Fig 4A and 4B). In contrast, all cSCC cell lines transfected with miR-135b mimics (to increase endogenous miR-135b expression) demonstrated further downregulation of LZTS1 (PM1–1.00 ± 0.11-fold; MET1–1.60 ± 0.15-fold; MET4–1.77 ± 0.07-fold) within 24 hours post-transfection when compared to the negative control as shown in Fig 5A and 5B. Collectively, these data demonstrate that LZTS1 expression is regulated by miR-135b in cSCC cells *in vitro*.

### miR-135b affects cSCC migration and invasion

**Fig 6:**
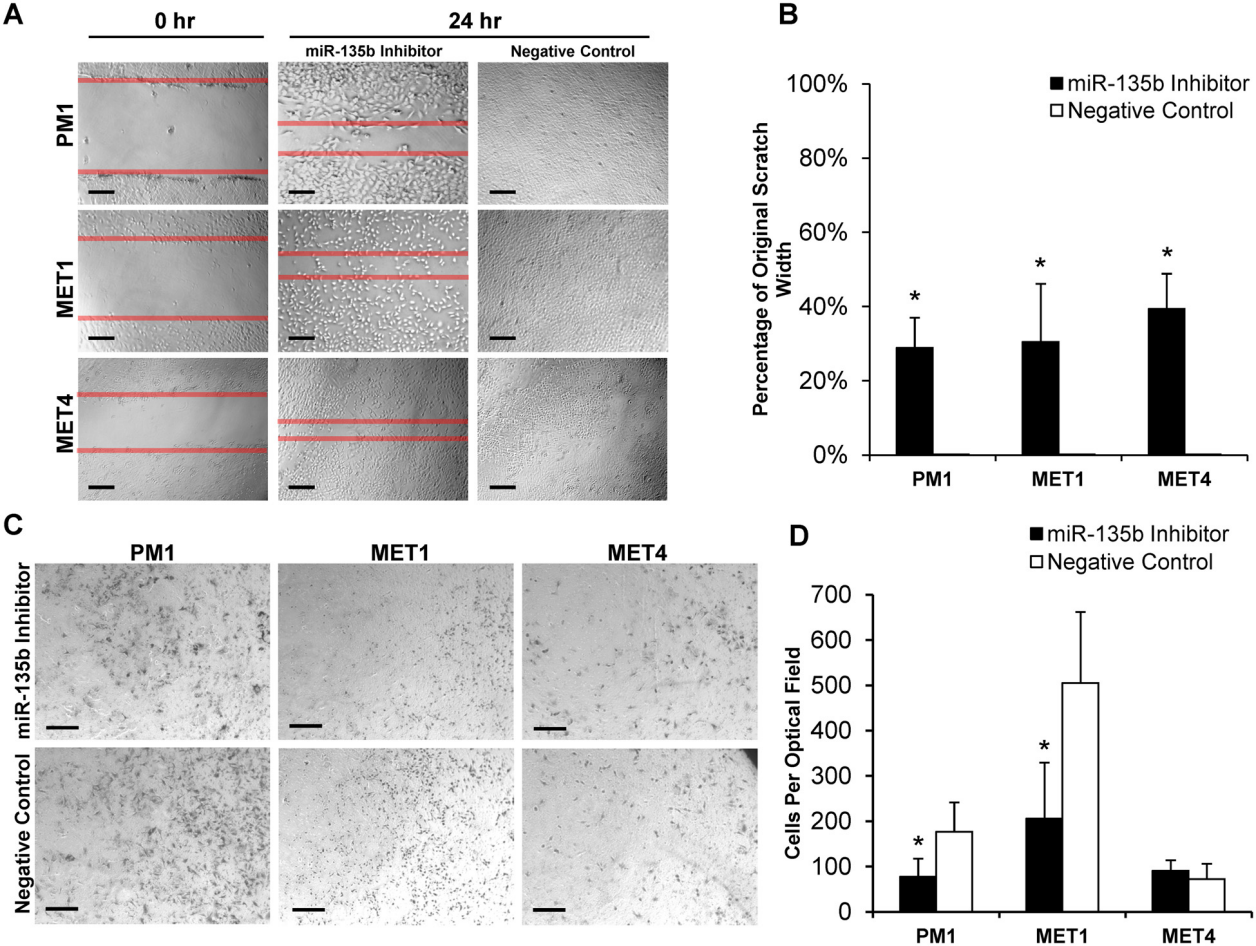
miR-135b inhibition delays cell migration and invasion in MET1, PM1 and MET4 cSCC cell lines. A, B) miR-135b inhibitor significantly delays cSCC cell migration in the wound scratch assay within 24 hours post-transfection. C, D) miR-135b inhibitor significantly reduces cSCC cell invasion in the Matrigel transwell invasion assay. * indicate p values less than 0.01. A) *Bar = 100um*; B) *Bar = 200um*.

**Fig 7:**
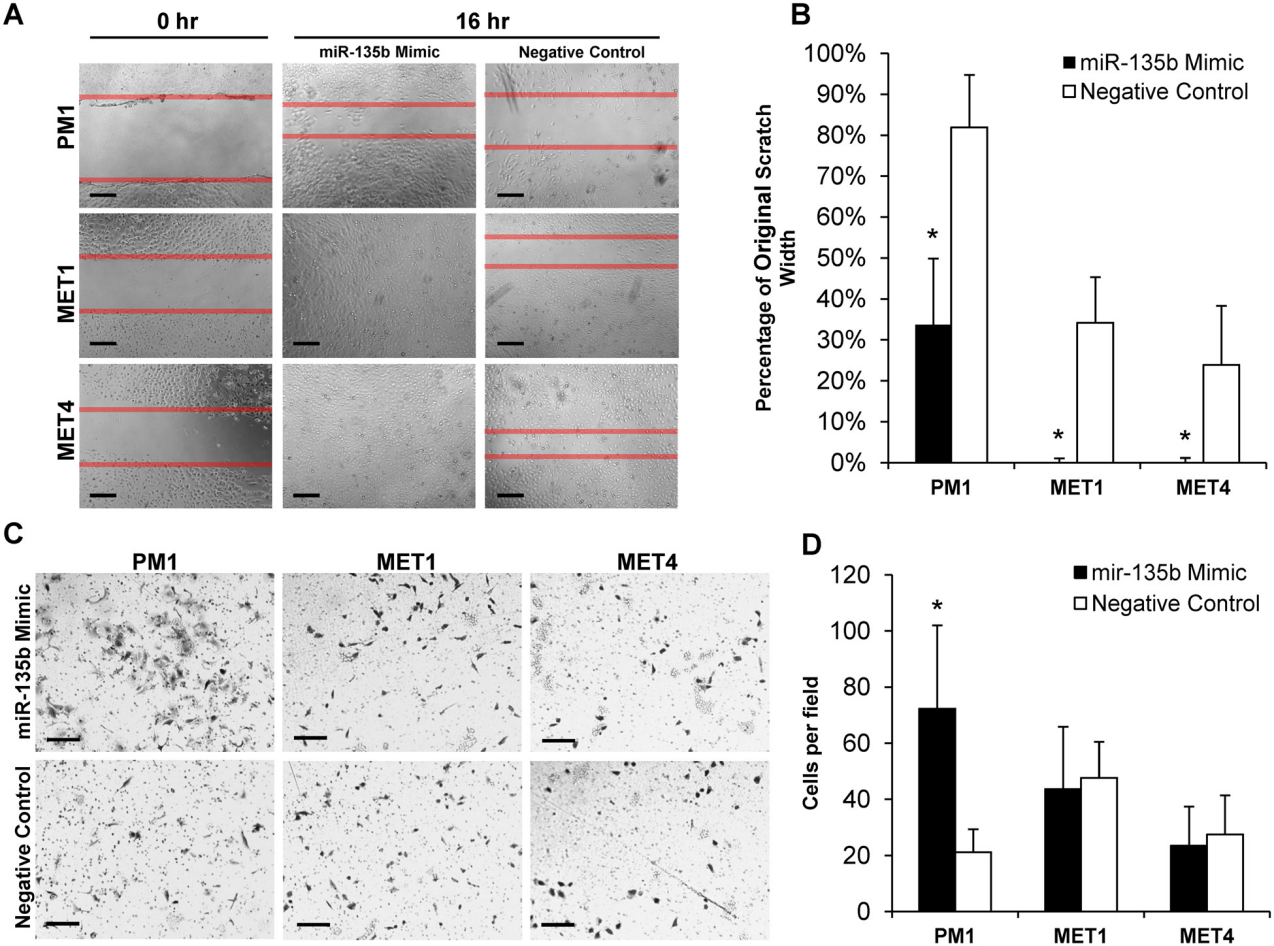
miR-135b overexpression increases cell migration and invasion in MET1, PM1 and MET4 cSCC cell lines. A, B) miR-135b mimic significantly increases cSCC cell migration in the wound scratch assay within 24 hours post-transfection. C, D) miR-135b mimic significantly increases cSCC cell invasion in the Matrigel transwell invasion assay in PM1 cell line. * indicate p values less than 0.01. A) *Bar = 100um*; B) *Bar = 200um*.

Since LZTS1 has been reported as a biomarker of poor clinical outcome in other tumor types [24, 30–32], we hypothesized that miR-135b could influence the metastatic invasiveness of cSCC by modulating LZTS1expression. To test this hypothesis, we examined the effects of miR-135b inhibition on cSCC cell migration and invasion, two key components of malignant tumor progression [33]. As shown in Fig 6A and 6B, treatment with miR-135b inhibitor significantly suppressed motility of all PM1 (29 ± 8%; *p*<0.001), MET1 (30 ± 16%; *p*<0.001), and MET4 (39 ± 9%; p<0.001) cell lines in a wound scratch assay compared with the negative control. Next we assessed the effect of miR-135b inhibition on cell invasion. Interestingly, miR-135b inhibition reduced cSCC invasion by 2.2-fold (*p*<0.01) in PM1 and by 2.4-fold (*p*<0.01) in MET1 cells when compared with the negative control (Fig 6C and 6D). The MET4 (metastatic) cell line invasive capacity was not affected by miR-135b inhibition. In contrast, miR-135b overexpression by synthetic miR-135b mimic further increased cancer cell motility (Fig 7A and 7B) and invasiveness (Fig 7C and 7D). These data indicate that miR-135b can regulate cell migration and tumor invasiveness in early stages of cSCC progression and can act as an oncogenic miRNA in human keratinocytes.

### Inhibition of miR-135b does not affect the cSCC proliferation

**Fig 8:**
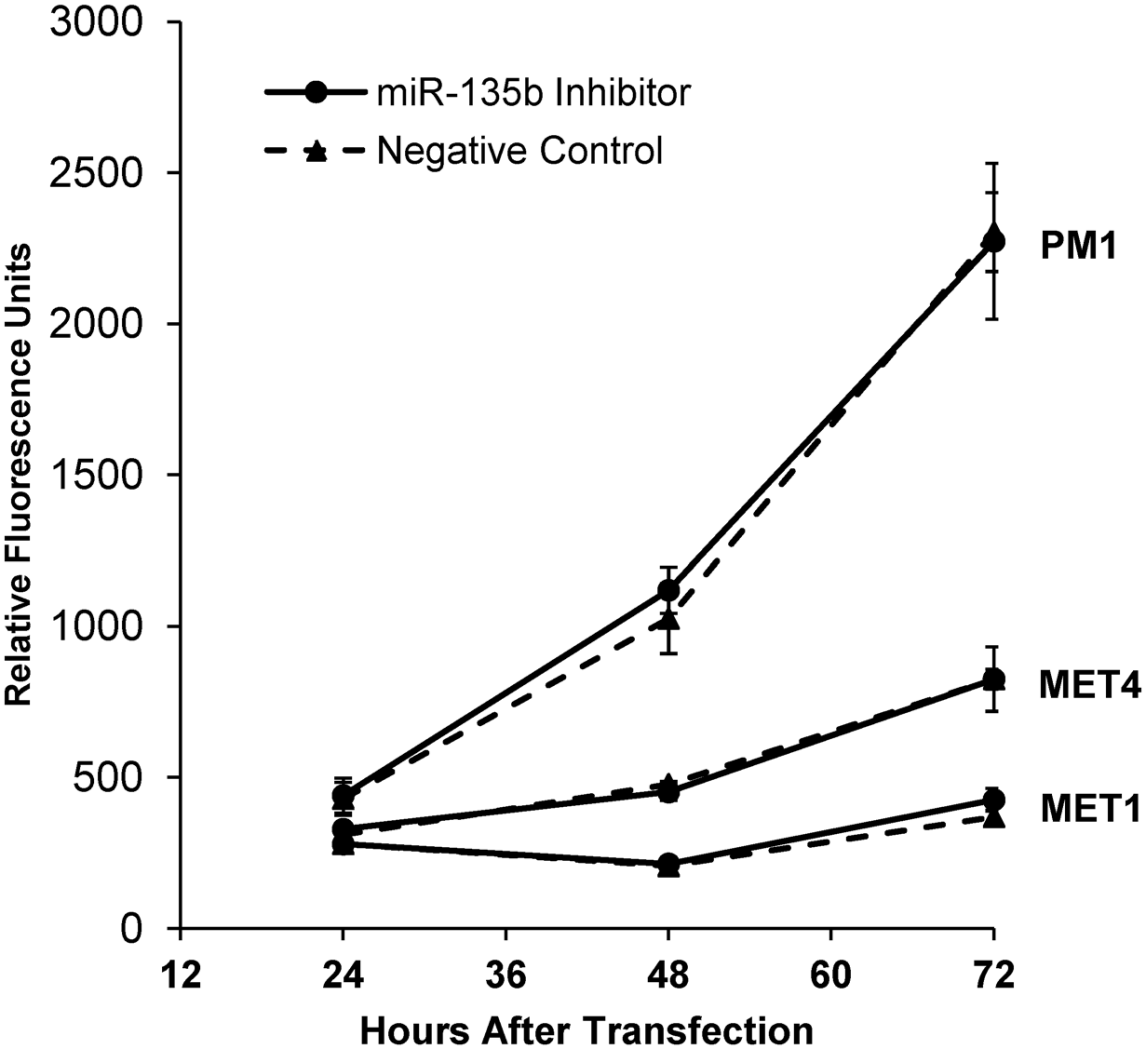
miR-135b does not affect the cSCC cell proliferation *in vitro*. Proliferation of cSCC cells transfected with miR-135b inhibitor *versus* negative control oligonucleotides. Each data point was obtained as an average of triplicate samples. P value from two-sided Student's *t* test compares the measurement of cellular DNA content via fluorescent dye binding between the two groups. p value greater than 0.05 was calculated for all three cell lines at all individual time points.

To determine if miR-135b affects the proliferative potential of cSCC cells, we performed proliferation assay on all three cSCC cell lines transfected with miR-135b inhibitor. Interestingly, none of the tested cSCC cell lines showed decreased proliferation capacity after the miR-135b inhibition when compared to negative control transfected cells as shown in Fig 8. Collectively these data indicate that inhibition of miR-135b blocks cSCC cell motility and invasiveness without inhibition of tumor cell proliferation, thus implicating involvement of additional pathways supporting the tumor growth.

## Discussion

The risk of developing cSCC is markedly increased in OTRs compared to the normal population. The sun exposure and the immunosuppressive regimen are important risk factors for the development of cSCC in OTRs. Recent studies suggest that some miRNAs are critical not only in promoting successful transplant outcomes after both solid organ and hematopoietic stem cell transplantation [34, 35], but can play an important role in tumor progression and metastasis. A genome-wide sequencing study demonstrated vast differences in gene expression profiles between cSCC, actinic keratoses and normal skin from immunosuppressed OTRs thus bringing to attention other molecular mechanisms such as miRNA alterations which may affect gene expression in this group of patients [36]. MiR-135b has been implicated in tumor progression of multiple cancer-types [14, 15, 37], but the status of miR-135b in cSCC from IC and OTRs and its functional role in either type of cSCC are currently unknown. Here, we demonstrated that miR-135b was the most elevated miRNA in cSCC tissues from both IC and OTR patients (Table 1A and 1B). There are several genes regulated by miR-135b, among them, a tumor suppressor gene, LZTS1 that was recently shown to be a miR-135b target-gene in multiple cancers, [15, 30, 31, 38], but had not been tested in cSCC. We found that inhibiting miR-135b in cSCC OTR-derived cell lines resulted in increased expression of LZTS1 transcript and protein levels (Figs 4 and 5). The resulting increase in LZTS1 levels suppressed cSCC cell migration (Fig 6A and 6B) and invasion (Fig 6C and 6D) without affecting cell proliferation, demonstrating that miR-135b is an oncomir that functions via downregulation of the LZTS1 tumor-suppressor gene. We also demonstrated that overexpression of endogenous miR-135b in all three cSCC cell lines using specific miR-135b mimic further downregulated the mRNA LZTS1 expression and increased tumor cell motility and invasion.

In our study the mRNA levels of miR-135b correlated with malignant cell phenotype. The metastatic cell line (MET4) had higher endogenous miR-135b expression level when compared to a cSCC cell line derived from dysplastic skin or primary tumor suggesting, that miR-135b may be one of the regulating factors in the tumor progression. Similarly, studies in colon cancer have demonstrated an increase in expression of miR-135b in both adenomas and carcinomas compared to normal epithelium [39]. The progressive increase in miR-135b expression from normal to polyp to carcinomatous tissues suggested that miR-135b dysregulation is an early event that is amplified with increasing dysplasia [13]. Thus data from our study suggest that miR-135b might similarly influence cSCC progression through modulation of tumor suppressors, such as LZTS1.

LZTS1 is a tumor suppressor gene [30, 31, 38] that is regulated by miR-135b in lung cancer [15], but the relationship and functional roles of the miR-135b/LZTS1 axis in cSCC were unknown. We found that LZTS1 expression was strongly downregulated in cSCC tumors compared with ubiquitous LZTS1 expression in normal epidermis (Fig 3). This fits with previous reports of decreased LZTS1 expression in multiple other tumor types e.g., lung, breast, prostate and kidney carcinomas [15, 23, 30–32, 40], in which LZTS1 levels were negatively correlated with tumor progression [24]. However, we did not find a significant correlation between LZST1 staining intensity and tumor grade in OTRs versus IC samples, presumably due to the low number of tumor samples from OTRs. We found that LZTS1 levels negatively correlated with miR-135b expression and malignant phenotype of cSCC cell lines (Fig 4). Importantly, miR-135b blockade increased LZTS1 expression resulting in decreased cSCC motility and invasion (Fig 7).

It has been shown that human tumor suppressor genes often function as negative regulators of the cell cycle. The down regulation of LZTS1 by upregulated miR-135b can lead to failure of cell cycle machinery in M-phase resulting in accumulation of genetic changes which often leads to genetic instability and aneuploidy [41]. This concept is in line with the fact that mice knockout for LZTS1develop a wide variety of tumors suggesting that aneuploidy phenotype could drive cellular transformation in vivo [21, 42]. Interestingly, our data demonstrate that inhibition of miR-135b does not affect proliferative capacity of cSCC in vitro indicating that loss of miR-135b while affecting the malignant cell migration and invasive functions, does not contributes to the tumor growth. In line with our results, in a recently published study Wu et al [43] describe regulation of metastasis suppressor-1 (MTSS1) by miR-135b in the colorectal cancer. MTSS1 may exert its function by acting as a scaffold protein that interacts with actin-associated proteins thus affecting lamellipodium formation and cell motility without affecting cell proliferation capacity.

Collectively, these data suggest that the miR-135b/LZTS1 signaling axis plays an important role in cSCC progression and suggests that miR-135b might be useful therapeutic target for cSCC not only in IC but in OTR patients. Further studies using the miR-135b interference strategy will be needed to develop new treatment modalities for this common human cancer.
